# Prognostic Value of Non-Invasively Determined Right Ventricular–Arterial Coupling Surrogate Parameters in Patients with Dilated Cardiomyopathy

**DOI:** 10.3390/jcm15062239

**Published:** 2026-03-16

**Authors:** Maria Iovănescu, Diana-Ruxandra Hădăreanu, Despina Toader, Oana Munteanu-Mirea, Ionuț Donoiu

**Affiliations:** 1 Department of Cardiology, University of Medicine and Pharmacy of Craiova, 200349 Craiova, Romaniaionut.donoiu@umfcv.ro (I.D.); 2 Department of Cardiology, Emergency County Hospital, 200642 Craiova, Romania; despinamtoader@yahoo.com

**Keywords:** cardiomyopathy, dilated, heart failure, ventricular function, right, echocardiography, echocardiography, three-dimensional, echocardiography, speckle tracking, pulmonary hypertension, hemodynamics, prognosis, risk assessment

## Abstract

**Background/Objectives**: Right ventricular dysfunction is frequent in patients with dilated cardiomyopathy (DCM) and contributes significantly to prognosis. This study evaluated the prognostic value of echocardiography-determined surrogate parameters of right ventricular–arterial (RV–PA) coupling in patients with DCM. **Methods**: A total of 88 patients admitted between January 2019 to September 2023 were retrospectively and prospectively assessed and followed for a mean of 14 months. The primary endpoint was rehospitalization for decompensated heart failure (HF); the secondary endpoint was all-cause mortality. The parameters studied included TAPSE/PASP, RVFAC/PASP, RVFWLS/PASP, and RVEF/PASP. **Results**: In univariate analysis, all indices were associated with rehospitalization, but multivariate analysis retained only RVFWLS/PASP and RVEF/PASP as independent predictors. Optimal cut-offs were identified as 1.2 for RVEF/PASP (sensitivity 72%, specificity 80%) and 0.46 for RVFWLS/PASP (sensitivity 72%, specificity 76%). None of the parameters correlated significantly with all-cause mortality. **Conclusions**: These findings highlight the prognostic utility of non-invasively derived RV–PA coupling indices for rehospitalization risk stratification in DCM.

## 1. Introduction

Large-scale studies have repeatedly demonstrated that the degree of left ventricle (LV) dilation and especially the severity of LV systolic dysfunction expressed by reduced LV ejection fraction (LVEF) play a major role in determining the prognosis of patients with dilated cardiomyopathy (DCM) [1,2]. In recent years, however, focus has shifted to the impact of right ventricle (RV) dysfunction in this category of patients, which has also proven to be significant. The percentage of patients with DCM who develop right ventricular dysfunction reaches, in some studies, up to 65% [3,4,5]. Although RV dysfunction is based on complex, often intricate, mechanisms, represented by its volume/pressure overload or even an intrinsic right ventricular myopathic process, most often, remodeling and subsequent reduction in RV function occur in the context of pulmonary hypertension (PH), secondary to left heart pathology [6].

Currently, the assessment of RV function benefits from a multimodal multiparametric imaging approach [7], and transthoracic echocardiography (TTE), although not the gold standard, is the first-line imaging approach used for investigation. Most echocardiographic parameters that evaluate RV function, despite having a high accuracy, are volume-dependent and can therefore over- or underestimate RV performance [8]. On the other hand, the relationship between RV contractility and afterload (right ventricular–arterial coupling), defined as the ratio of end-systolic elastance (Ees) to arterial elastance (Ea), optimally reflects the RV mechanical work–oxygen consumption balance [9,10].

The gold standard for measuring RV and pulmonary artery (PA) coupling indices is right heart catheterization with pressure–volume curves. This investigation is not routinely performed, and thus, the need for parameters that reflect right ventricular–arterial coupling that can be determined non-invasively arose. Several echocardiographic surrogate parameters have been proposed: TAPSE/PASP, RVFWLS/PASP, RVFAC/PASP, RVEF/PASP, RVSV/RVESV (TAPSE—tricuspid annular plane systolic excursion, PASP—pulmonary artery systolic pressure, RVFWLS—right ventricular free wall longitudinal strain, RVEF—right ventricular ejection fraction, RVSV—right ventricular stroke volume). Among these, TAPSE/PASP has been validated as a relevant non-invasive parameter for measuring RV–pulmonary arterial circulation coupling, showing a prognostic role in patients with severe pulmonary artery hypertension (PAH) (with a proposed cut-off value of 0.31 mm/mmHg) [11]. The prognostic importance of these indices in terms of the risk of rehospitalization or death has not been studied extensively in the pathology that is the subject of this research. While previous studies have evaluated surrogate RV-PA coupling indices such as TAPSE/PASP or RVEF/PASP, most were conducted in heterogeneous populations, including PAH or mixed-etiology heart failure. Data focusing specifically on non-ischemic DCM remain scarce.

Thus, the main objectives of the study were: (i) to evaluate the prognostic value of surrogate parameters of right ventricular–arterial coupling determined non-invasively by TTE, namely: TAPSE/PASP, RVFWLS/PASP, RVFAC/PASP, and RVEF/PASP in patients with DCM, and (ii) to identify their cut-off values associated with the endpoints, namely rehospitalization for decompensation of heart failure (HF) or death from any cause.

## 2. Materials and Methods

### 2.1. Study Design

The study was a retrospective and prospective study and included patients diagnosed with DCM who were admitted to the Cardiology Clinic of the Craiova County Emergency Clinical Hospital, Romania between January 2019 and September 2023. The inclusion criteria were: (i) the diagnosis of DCM based on LV dilation and systolic dysfunction (with or without the presence of dilation and/or RV dysfunction); LV dilation was assessed by the presence of an indexed LVEDV of at least 75 mL/m^2^ in men or 62 mL/m^2^ in women, calculated by TTE and global LV systolic dysfunction by an LVEF below 50% [12,13,14]; (ii) age over 18 years; (iii) written informed consent from each patient.

The exclusion criteria were: (i) the presence of significant coronary artery disease (epicardial coronary artery stenoses of at least 70%) that could have accounted for the LV dilation and global systolic dysfunction; (ii) unsatisfactory quality of echocardiographic acquisitions; (iii) absence of a euvolemic status at the time of the echocardiographic evaluation, (iv) presence of severe tricuspid regurgitation. These exclusion criteria were chosen to reduce major confounders in the evaluation of RV–PA coupling. Severe tricuspid regurgitation markedly alters RV loading conditions and affects the accuracy of PASP estimation, while coronary stenosis ≥70% was excluded to avoid overt ischemic cardiomyopathy, which follows different remodeling and prognostic pathways than non-ischemic DCM.

From the initial group of 120 patients, 32 were excluded (11 due to the presence of ischemic heart disease, 8 due to the suboptimal ultrasound window, and 13 due to significant congestion/severe RT). The final group consisted of 88 patients, of whom 50 were enrolled prospectively and 38 retrospectively. At the time of inclusion in the study, patients were evaluated clinically, biologically, and by conventional and advanced TTE. We followed them for an average period of 14 months. The primary endpoint in the study was rehospitalization for decompensated HF, and the secondary endpoint was death from any cause. For survival analysis, we used the time from the first rehospitalization for decompensated HF to the time of death.

The study was approved by the Ethics Committee of the University of Medicine and Pharmacy of Craiova, Romania (registration number 160/24.09.2021) and complied with the ethical principles of the Declaration of Helsinki.

### 2.2. Transthoracic Echocardiography

For the echocardiographic examination of patients, we used a Vivid E95 ultrasound machine (GE, Vingmed, Akershus, Norway). We acquired images after the systemic and/or pulmonary congestion phenomena had resolved, and after measuring blood pressure (BP), heart rate (HR), height, weight, and BSA in all patients. We initially performed conventional echocardiography (two-dimensional echocardiography—2DE, M-mode, and Doppler), later advanced two-dimensional speckle-tracking echocardiography (2DSTE), and three-dimensional echocardiography (3DE). We performed the analysis offline using the EchoPAC version 204 system (GE Vingmed Ultrasound, Horten, Norway).

#### 2.2.1. Two-Dimensional Speckle-Tracking Echocardiography (2DSTE)

For the analysis of LV deformation, we calculated global longitudinal strain (GLS) using the 17-segment model. Initially, we acquired dedicated LV images—apical four-chambers (A4C), apical two-chambers (A2C), and apical long axis with optimal temporal resolution. We defined the region of interest (ROI) after drawing the LV endocardial and epicardial borders. We modified the images automatically generated by the software where we deemed necessary. We calculated the myocardial work parameters derived from the LV GLS: global work index (GWI), global constructive work (GCW), global work waste (GWW), global work efficiency (GWE) [15]. For the RV, we calculated the RVFWLS using the A4C view focused on the RV and following the same principles of acquisition, definition of ROI, and adjustment of images. For the left atrium (LA), we initially acquired images of A4C and A2C views in which the LA had the largest size and was visualized entirely throughout the cardiac cycle. After tracing the endocardium, we used a dedicated atrial analysis mode and adjusted or rejected the automatically generated ROI, depending on the accuracy of the recording. We calculated LA strain in the reservoir, conduit, and contraction phases of the LA cycle. We defined the zero-strain reference in the LV end-diastole. For the right atrium (RA), we acquired A4C images focused on the RV and followed the same principles.

#### 2.2.2. Three-Dimensional Echocardiography (3DE)

For the 3D acquisitions, we used a dedicated probe (M4V, GE, Vivid E95, Vingmed). We first optimized the 2D images and performed multibeat acquisitions, incorporating the structures of interest to have an optimal resolution, in apnea to avoid the appearance of artifacts. For the LV, we used apical sections, standard or modified. For the RV, we used apical views focused on the RV (so that it was included in its entirety). We used a semi-automatic algorithm (4D autoLVQ and 4D autoRVQ, respectively) to quantify the volumes and the LV/RV ejection fraction. We correctly aligned the sections, set the landmarks, checked the frames defined by the algorithm as end-diastolic and end-systolic, respectively, and the initial contours generated semi-automatically. We checked the 3D model and adjusted where the algorithmic detection was unsatisfactory. For the 3DE acquisition of the atria, we followed the same principles, starting from the optimization of the 2DE images to include the entire atrium in the analysis. For LA, we also used a semi-automatic algorithm (4D auto LAQ). We obtained the atrial volumes and the atrial emptying fraction.

#### 2.2.3. RV Systolic Function Parameters

To assess RV function, we measured the following parameters:

- TAPSE: to assess the systolic movement of the tricuspid annulus towards the RV apex (and thus the longitudinal RV systolic function at baseline), we used M-mode at the lateral tricuspid annulus [13].
- RVFAC: to calculate the percentage change in RV area (and thus both longitudinal and radial RV shortening), we measured RV areas in end-diastole and end-systole in RV-focused A4C [16].
- RVFWLS: we used 2DSTE to evaluate the deformation of only the RV free wall in the A4C section focused on the RV [16]. We chose not to calculate the RV GLS because it involved including the interventricular septum in the analysis, which is a common wall of the 2 ventricles.
- RVEF: we used 3DE to calculate volumes and ejection fraction according to the described acquisition protocol [17].

#### 2.2.4. Estimation of Pulmonary Arterial Systolic Pressure

To estimate PASP, we first calculated the maximum RV-RA gradient based on the maximum tricuspid regurgitation jet velocity measured on the CWD envelope using the simplified Bernoulli equation: RV-RA gradient = 4 × (maximum tricuspid regurgitation jet velocity)^2^. We then estimated the RA pressure based on the diameter of the inferior vena cava and its degree of collapse during inspiration. Finally, we obtained PASP as the sum of the maximum RV-RA gradient and the RA pressure.

#### 2.2.5. Surrogate Parameters of Right Ventricular–Arterial Coupling

To estimate the coupling between the RV and the pulmonary arterial circulation, we used some of the parameters proposed in various other studies, namely: TAPSE/PASP, RVFAC/PASP, RVFWLS/PASP, and RVEF/PASP.

#### 2.2.6. Analysis of Variability

We assessed inter- and intraobserver variability by reanalyzing 10 random data sets using specific correlations.

### 2.3. Statistical Analysis

We performed the statistical analysis using the SPSS program, version 28.0.1.1. To verify the normal distribution of the variables, we used the Kolmogorov–Smirnov test. We expressed the normally distributed continuous variables as mean ± standard deviation. We expressed the categorical variables numerically and in percentages. In the first stage of the analysis, we divided the initial group of patients into two groups, depending on the presence or absence of rehospitalizations for HF decompensation as the primary endpoint.

We compared the data of the 2 groups using the T-test for independent variables and the Mann–Whitney U test, depending on the distribution of the variables. We compared the categorical variables using the Fisher test. To analyze the prognostic impact of the right ventricular–arterial coupling surrogate parameters determined by echocardiography, we performed Cox regressions. Initially, the analysis was univariate (performed on both RV-AP coupling indices and other echocardiographic parameters with a potential or proven prognostic role), and later we included in the multivariate analysis statistically significant parameters with a proven prognostic role. To express the results, we used hazard ratios (HR) with 95% confidence intervals. Pairwise correlations between RV parameters were assessed using Spearman coefficients. Variance inflation factors were calculated to confirm the absence of significant multicollinearity. Proportional hazards assumptions were evaluated using time–covariate interaction terms for the variables included in the multivariate Cox model. The parameters that had prognostic value after the multivariate analysis were included in an ROC (receiver operating characteristic) curve. Based on the highest sum of sensitivity and specificity, we chose the cut-off values of the respective parameters for the prediction of the primary endpoint. Given the limited sample size, the ROC-derived cut-offs were not internally validated through resampling procedures such as bootstrapping. Then, we performed a Kaplan–Meier survival analysis and used the log-rank test to compare the survival curves. The results were considered to have statistical significance at a value of *p* < 0.05. The second stage of the statistical analysis consisted of dividing patients into 2 groups according to the presence or absence of the secondary endpoint, namely, death from any cause. We used the same tests mentioned above to compare the data between the groups.

## 3. Results

### 3.1. Primary Endpoint: Rehospitalization for Decompensated HF

#### 3.1.1. Characteristics of the Study Population

Of the 88 patients included in the study, 84.6% were male, and the mean age was 56.9 ± 10 years. They were followed for a mean period of 14 months for rehospitalizations due to decompensated HF. During the follow-up period, 55% of the patients suffered at least one rehospitalization for HF. Initially, the group was divided into two groups, depending on the presence or absence of the primary endpoint. There were no significant differences between the two groups in terms of age, BSA, HR, but BP values were lower in the group that achieved the primary endpoint. NT-proBNP values were significantly higher in patients who had at least one rehospitalization, and the NYHA class was more advanced. Regarding the presence of comorbidities such as hypertension, diabetes, dyslipidemia, or tobacco use, their frequency was comparable between groups (Table 1).

At baseline, all patients were receiving guideline-directed medical therapy according to contemporary heart failure recommendations, with no significant differences in the major classes of HF treatment between groups (Table 2).

#### 3.1.2. Conventional and Advanced Echocardiography

There were no statistically significant differences between the LV-indexed volumes measured by 3DE (i.e., LVEDV, LVESV, LV SV) between patients who reached the primary endpoint (rehospitalization for decompensated HF) and those who did not reach the primary endpoint. LV systolic function assessed by both 3D LVEF and LV GLS instead was more impaired in patients who were rehospitalized versus those who did not undergo rehospitalization (3D LVEF 26.8 ± 8% versus 33.1 ± 10%, *p* = 0.01, LV GLS 6.4 ± 3% versus 8.1 ± 3%, *p* = 0.02). LV filling pressures estimated by the mean E/E’ ratio had comparable values between groups (*p* = 0.2). Regarding LA volumes and function, the minimum indexed LA volume determined by 3DE was higher in patients who reached the primary endpoint (44.3 ± 16 mL/m^2^ versus 35.3 ± 13 mL/m^2^, *p* = 0.04), and the emptying fraction was lower (18.7 ± 19%, *p* = 0.01). However, the LA biplane strain during the reservoir period (2DSTE) had comparable values between groups (*p* = 0.2) (Table 3).

Patients who underwent rehospitalization had significantly higher indexed RV volumes measured by 3DE (indexed RV EDV 70.3 ± 28 mL/m^2^ versus 50.7 ± 24 mL/m^2^, *p* = 0.01, indexed RVESV 47.4 ± 21 mL/m^2^ versus 27.1 ± 15 mL/m^2^, *p* = 0.001). All echocardiographic parameters (classical and advanced) used to evaluate RV function had significantly lower values in the group that reached the primary endpoint (TAPSE 15.2 ± 3 versus 17.9 ± 4 mm, RVFAC 31 ± 10 versus 39.7 ± 6%, RVFWLS 13.1 ± 6 versus 17.6 ± 5%, RVEF 36.7 ± 9 versus 46.7 ± 7%, *p* ≤ 0.001 for all). Also in this group, the maximum and minimum volumes of RA indexed were higher (RA maximum volume 46.9 ± 21 versus 32.2 ± 13 mL/m^2^, *p* = 0.008, RA minimum volume 37 ± 19 versus 23.5 ± 11 mL/m^2^, *p* = 0.006), and the function parameters of AD, namely the strain in the reservoir period (2DSTE) and the emptying fraction (3DE) were significantly lower (RASr: 12.5 ± 10 versus 19.3 ± 10%, *p* = 0.009, RA emptying fraction: 22.3 ± 12 versus 30.3 ± 13, *p* = 0.02). The estimated systolic pulmonary artery pressure was significantly higher in the group of patients who were rehospitalized compared to the group without rehospitalizations (PASP 40 ± 15 versus 28 ± 14 mmHg, *p* < 0.001) (Table 4).

Regarding the surrogate RV-PA coupling parameters and their impact on prognosis, all indices obtained noninvasively by TTE and which were proposed to reflect the relationship between RV function and pulmonary arterial circulation had significantly lower values in patients who had at least one rehospitalization for HF decompensation (TAPSE/PASP 0.46 ± 0.2 versus 0.82 ± 0.4 mm/mmHg, *p* = 0.002, RVFAC /PASP 1 ± 0.8 versus 1.79 ± 0.9, *p* = 0.001, RVFWLS/PASP 0.44 ± 0.4 versus 0.81 ± 0.5, *p* = 0.002, RVEF/PASP 1.1 ± 0.8 versus 2.1 ± 1. *p* < 0.001) (Table 5).

**Figure 1:**
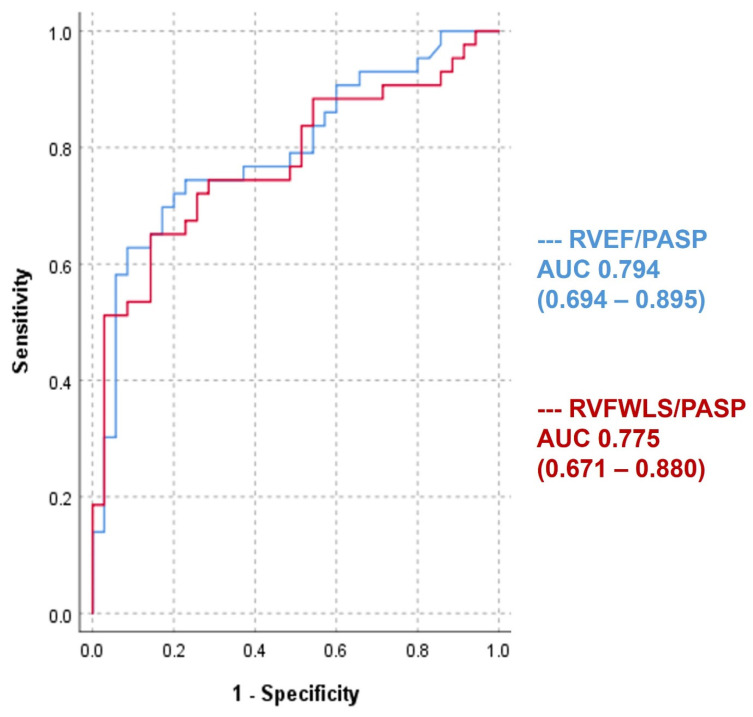
ROC and AUC analysis for RVEF/PASP and RVFWLS/PASP.

In the univariate Cox analysis, we demonstrated that each of TAPSE/PASP, RVFAC/PASP, RVFWLS/PASP, and RVEF/PASP influences prognosis and, in the case of the present study, rehospitalizations for HF decompensation. We also performed univariate regression for the RV function parameters (TAPSE, RVFAC, RVFWLS, RVEF) and LV (LV GLS, LVEF). In the multivariate Cox analysis, we added the latter to see if the RV-AP coupling parameters maintain their influence on prognosis when combined in a multivariate model. This multivariate model also included TAPSE, RVFAC, RVFWLS, RVEF, LVEF, and LV GLS. In multivariate regression, only RVFWLS/PASP and RVEF/PASP remained independent predictors of rehospitalization (*p* = 0.002 and 0.001, respectively) (Table 6). As shown in Supplementary Tables S1 and S2, RV parameters demonstrated only moderate intercorrelations and acceptable variance inflation factor values. We then introduced these two parameters into the ROC analysis (Figure 1), where it was shown that RVEF/PASP has a better ability than RVFWLS/PASP to predict rehospitalizations (AUC—area under the curve; RVEF/PASP 0.794 (0.694–0.895) and RVFWLS/PASP 0.775 (0.671–0.880)).

**Figure 2:**
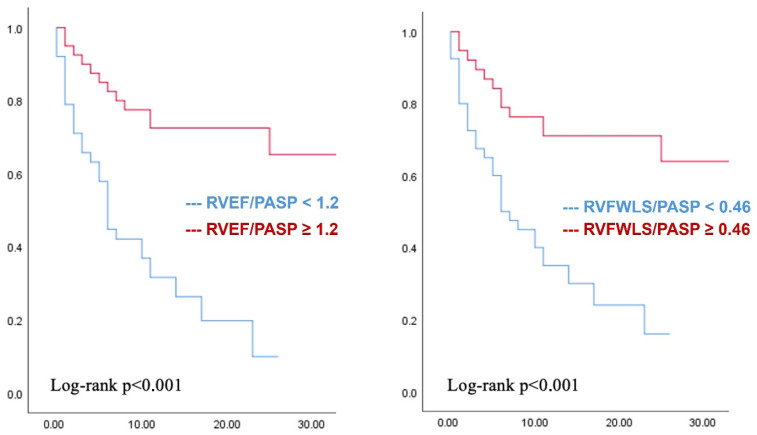
Kaplan–Meier survival curves according to the proposed cut-offs for RVEF/PASP and RVFWLS/PASP.

Based on the sum of the highest sensitivity and specificity, we chose the optimal cut-off values of the two parameters to predict rehospitalizations: RVEF/PASP = 1.2 (sensitivity 72%, specificity 80%), RVFWLS/PASP = 0.46 (sensitivity 72%, specificity 76%). Using these cut-offs, we generated survival curves with Kaplan–Meier (Figure 2). Subjects with values of RVFWLS/PASP < 0.46 and RVEF/PASP < 1.2 had a more unfavorable evolution, higher risk of rehospitalization, and shorter time until reaching the primary endpoint.

### 3.2. Secondary Endpoint: Death from Any Cause

During the follow-up period, 23% (20) of patients died (100% men, mean age 59.9 ± 10 years). Although patients who died were older (59.9 ± 10 versus 56.1 ± 11 years), more frequently dyslipidemic, hypertensive, and smokers, statistically, the differences were not significant (*p* > 0.05 for all). NT-proBNP values were significantly higher in the group that reached the endpoint (*p* = 0.01) (Table 7).

When comparing the RV-PA coupling surrogate parameters between the two groups, the values did not differ with statistical significance, and none of them was associated with death from any cause (Table 8).

## 4. Discussion

Through this study, we investigated echocardiographic parameters of right ventricular–arterial coupling in patients with DCM. We demonstrated the following:

1. Right ventricular–arterial coupling assessed non-invasively by surrogate echocardiographic parameters is significantly more impaired in patients with DCM who undergo rehospitalization for decompensation of HF.
2. Although in univariate analysis all four investigated indices (TAPSE/PASP, RVFAC/PASP, RVFWLS/PASP, and RVEF/PASP) proved to be predictors of rehospitalization, following multivariate analysis, only RVFWLS/PASP and RVEF/PASP remained independent predictors of rehospitalization.
3. RVEF/PASP has a better ability than RVFWLS/PASP to predict rehospitalization.
4. The proposed optimal cut-off values of the two parameters to predict rehospitalizations were RVEF/PASP = 1.2 (sensitivity 72%, specificity 80%), RVFWLS/PASP = 0.46 (sensitivity 72%, specificity 76%).
5. Tight ventricular–arterial coupling surrogate parameters are not associated with death from any cause in patients with DCM.

RV function is conditioned by two elements, namely: its volume or pressure overload, respectively intrinsic contractile dysfunction, the consequence of a right ventricular myopathic process. In the case of DCM, any of these conditions (volume overload—severe tricuspid regurgitation, pressure overload—pulmonary hypertension, or intrinsic RV myopathy) can occur; moreover, they are not mutually exclusive, but, on the contrary, can coexist, leading to the installation of RV dysfunction. Secondary PAH in DCM is based on a complex relationship between the left ventricle, mitral valve, and left atrium that culminates in the retrograde transmission of increased pressures in the pulmonary arterial bed and the occurrence of pulmonary vascular remodeling [18,19,20].

The RV response to chronic increase in afterload comprises a first, homeometric phase, in which its wall thickness increases compensatory to reduce parietal stress, and its volumes and function remain within normal limits. Thus, a “coupling” of it with the pulmonary arterial circulation occurs, with the aim of maintaining an optimal blood flow, therefore a minimally modified stroke volume in the context of increased RV contractility. In the second phase, called heterometric, a maladaptive remodeling occurs, with progressive RV dilation and dysfunction [6,21,22]. The consequence is a decrease in stroke volume, increased parietal stress, and right ventricular–arterial “uncoupling” [22].

The gold standard for evaluating this relationship is right heart catheterization with the acquisition of pressure–volume curves and the ratio between end-systolic elastance (Ees) and arterial elastance (Ea). Ees reflects RV contractility and is a load-independent parameter, and Ea reflects arterial function, independent of RV pump function. Since right heart catheterization is not routinely performed, echocardiography remains the basic investigation for assessing both RV function and its relationship with pulmonary arterial circulation. There are several echocardiographic parameters used to quantify RV function, conventional and advanced—TAPSE, myocardial velocity measured at the lateral tricuspid annulus (S-wave), Tei index, RVFAC, RVFWLS, and RVEF. Most have proven their prognostic role in DCM, but they are not infallible. TAPSE, S-wave, and RVFAC are volume-dependent and do not evaluate global RV function. RVFWLS and RVEF depend more on image quality, postprocessing, and operator experience [8,23,24,25,26,27,28]. By relating these parameters to the estimated PASP, we can assess the relationship between RV contractility and noninvasive afterload. The TAPSE/PASP parameter has been validated as a surrogate parameter of RV-PA coupling [11].

The aim of the present study was to investigate the importance of the aforementioned indices on the prognosis of patients with DCM. The primary endpoint was rehospitalization for decompensated HF, and the secondary endpoint was death from any cause. Regarding the primary endpoint, in univariate analysis, all four surrogate parameters investigated correlated with rehospitalizations. Given that both echocardiographic parameters for RV function and those for LV function (specifically LV GLS and LVEF) had lower values in the group of patients who reached the primary endpoint, and their prognostic significance has been proven in various studies, we then performed a multivariate analysis to see if TAPSE/PASP, RVFAC/PASP, RVFWLS/PASP, and LVEF/PASP maintain their prognostic power. In multivariate regression, only RVFLWS/PASP and RVEF/PASP remained independent predictors of rehospitalization, while TAPSE/PASP and RVFAC/PASP lost their predictive role. This result is in contradiction with other studies dedicated to the RV-PA coupling in HF (154). Bosch et al. demonstrated that TAPSE/PASP correlated with the composite endpoint (death and rehospitalization for HF) in patients with chronic HF, both with preserved LVEF and with reduced LVEF [29]. In another study, Bragança et al. showed that TAPSE/PASP has the ability to predict the lack of response to resynchronization therapy in a cohort of pharmacologically optimally treated HF patients proposed for device implantation [30]. Guazzi et al. evaluated the predictive role of TAPSE/PASP combined with functional capacity (objectified by cardiopulmonary exercise testing) in a group of patients with HF. A low value of TAPSE/PASP (cut-off used 0.36 mm/mmHg) was found to be associated with reduced functional capacity and increased risk of adverse cardiac events [31]. On the other hand, Alexander Schmeisser et al. compared the prognostic relevance of TAPSE and TAPSE/PASP in a cohort of 110 HF patients with reduced LVEF and demonstrated that, although both TAPSE and TAPSE/PASP are independent predictors of adverse events, indexing TAPSE to PASP is not superior to measuring TAPSE alone [32].

The explanation for the result in the present study could be that TAPSE, although it is one of the most used parameters of RV function, only evaluates its longitudinal function at the level of the basal segment of the free wall, is dependent on volume, and is influenced by global cardiac motion [33]. It is therefore possible that TAPSE/PASP is not a very reliable surrogate parameter for estimating the RV-PA relationship.

As for RVFAC/PASP, to our knowledge, there are no studies that analyze this parameter as a surrogate of RV-PA coupling in patients with DCM and imply its prognostic role. Although RV FAC evaluates both longitudinal and radial shortening of the RV, it excludes the contribution of the RV ejection tract to its contractile performance and is also a volume-dependent parameter. Thus, the indexing of RVFAC to PASP could have limitations in estimating RV-PA coupling and, implicitly, in determining the prognostic role of the latter in multivariate regression. RVFLWS/PASP and RVEF/PASP remained independent predictors of rehospitalization even after the adjustment for cofactors in our study. RVFLWS assesses RV longitudinal function at the level of the entire free wall and is a parameter that is less volume-dependent and has a superior prognostic value compared to classical RV function parameters (TAPSE, RVFAC) [34,35,36]. RVFWLS/PASP was proposed as a new echocardiographic prognostic marker by Ünlü and colleagues in a study in which they demonstrated that it accurately and superiorly predicts all-cause mortality and the need for heart–lung transplantation in a population of patients with PAH (cut-off of 0.19) [37]. In another analysis conducted by Iacoviello, RVFWLS/PASP was shown to be independently associated with an increased risk of mortality in a cohort of patients with chronic HF undergoing conventional therapy, thus improving their prognosis stratification [38]. Finally, this ratio emerged as a valuable independent predictor of the outcome in HF and ventricular secondary mitral regurgitation [39].

Regarding the last RV function parameter indexed to PASP, namely RVEF, it is the most reliable in assessing RV contractile performance because it overcomes the limitations of previously described echocardiographic markers—geometric assumptions, angle/volume dependence, assessment of regional rather than global function, etc.—and has been validated in comparison with cardiac MRI, the gold standard for assessing biventricular volumes and function [40]. It has the highest ability to predict survival among all echocardiographic parameters of RV function and remains useful even after cardiac surgery [23,28,41,42]. The results of our study regarding the prognostic role of RVEF indexed to PASP are in agreement with other studies addressing this surrogate parameter. RFEV/PASP <0.44 as an expression of RV-PA uncoupling was associated with a poor prognosis in patients with PH in a retrospective study (167). Nochioka et al. also demonstrated that RVEF/PASP has the ability to predict hospitalization for HF or death in a cohort of patients with overt or subclinical HF [43].

The fact that in the present study, in the multivariate regression of the four surrogate markers of RV-PA coupling, only RVFWLS/PASP and RVEF/PASP remained independent predictors for rehospitalizations, and that, of these two, RVEF/PASP has a better ability to predict rehospitalizations, can be justified precisely by the superiority of RVFWLS but especially of RVEF’s ability to portray RV function. It is therefore not surprising that by indexing RVFWLS and RVEF to PASP (versus reporting TAPSE or RVFAC to PASP), we can identify patients with DCM with an increased risk of rehospitalization. Even after including RV function parameters (not indexed to PASP) and LV in the multivariate analysis, RVFWLS/PASP and RVEF/PASP retained their predictive capacity. These results emphasize the importance of assessing RV contractility and pulmonary arterial circulation status, not only separately but also in an integrated manner.

From a clinical perspective, the cut-offs identified in our study (RVFWLS/PASP < 0.46 and RVEF/PASP < 1.2) help to recognize patients with early RV–PA uncoupling who are at higher risk of recurrent decompensation, even when conventional RV parameters remain only mildly abnormal. In our daily practice, patients below these thresholds typically require closer follow-up, earlier optimization of guideline-directed medical therapy, and more frequent reassessment of volume status. For example, individuals with an RVFWLS/PASP value below 0.46 often show reduced functional reserve despite borderline TAPSE, prompting a more intensive follow-up strategy. Similarly, patients with an RVEF/PASP ratio below 1.2 usually demonstrate reduced capacity to adapt to increases in afterload and may benefit from earlier referral to specialized heart failure care. These examples illustrate how integrating RV–PA coupling indices into routine evaluation may strengthen risk stratification and support individualized management.

Right ventricular function depends on RV myocardial contractility and its degree of adaptation to pulmonary vascular resistance. Adequate coupling between RV and PA implies an increase in RV contractility with increasing afterload, so that RV function is maintained within normal ranges. RV-PA decoupling in the context of a chronic increase in afterload indicates an excess of RV adaptive capacity, thus a ventriculo-arterial mismatch and ultimately right ventricular failure [44]. Since the assessment of this complex interrelationship cannot be routinely performed invasively, the need arose to develop surrogate indices obtained non-invasively capable of providing valid information.

The clinical relevance derives from the fact that the development of RV-PA decoupling allows the identification of patients at risk, whose follow-up and management can be adapted and individualized with the aim of improving the prognosis. In the present study, RV-PA coupling assessed non-invasively did not correlate with the secondary endpoint, namely death from any cause in patients with DCM (which is in contrast to the results of other studies on echocardiographic parameters of RV-PA coupling and mortality in various cohorts of patients with cardiovascular pathologies) [29,37,38,43]. However, it correlated with rehospitalizations for HF decompensation, RVFWLS/PASP, and RVEF/PASP being independent predictors.

Although not all of these parameters are validated against right heart catheterization, and large-scale studies are needed in this regard, the results of our study support the fact that the combined assessment of RV and pulmonary arterial circulation is essential, as this approach can improve the risk stratification of patients with DCM and the accuracy in predicting their prognosis.

The absence of significant mortality associations may be explained by the low number of deaths and the relatively short follow-up period, which likely limited statistical power. Competing risks such as earlier rehospitalizations and treatment adjustments could also have influenced survival outcomes.

***Limitations***: The most important limitation is that this is a single-center study conducted on a relatively small cohort of patients with DCM (mostly men). The observed male predominance reflects the regional epidemiology of non-ischemic DCM rather than a selection bias, but we acknowledge that this imbalance may limit generalizability. Given the limited sample size, the ROC-derived cut-offs were not internally validated through resampling procedures, such as bootstrapping, which may increase the risk of overfitting. Also, patients included in the study did not benefit from right heart catheterization to validate the results obtained.

## 5. Conclusions

Through this study, we have evaluated right ventricular–pulmonary arterial coupling surrogate parameters non-invasively determined with TTE—mainly TAPSE/PASP, RVFWLS/PASP, RVFAC/PASP, and RVEF/PASP, as well as if and how they can influence DCM patients’ prognosis. The primary endpoint was rehospitalization for decompensated HF, and the secondary endpoint was death from any cause. We have proven that right ventricular–arterial coupling, non-invasively determined through these parameters, is significantly more impaired in patients suffering rehospitalizations for decompensated HF. Of all the aforementioned indices, in multivariate analysis, only RVFWLS/PASP and RVEF/PASP remain independent predictors of rehospitalizations. RVEF/PASP has the best ability to predict rehospitalizations. The proposed cut-off values to predict rehospitalization for the two parameters have been 1.2 for RVEF/PASP and 0.46 for RVFLWS/PASP. However, there is no association between right ventricular–arterial coupling surrogate parameters and death from any cause in DCM patients. The combined understanding and evaluation of the RV and arterial pulmonary circulation is extremely important because using this approach, compared to an isolated RV evaluation, helps better stratify DCM patients’ prognosis.

## Figures and Tables

**Table 1:** Characteristics of the studied population.

Variable	All Pts (n = 88)	Rehospitalization + (n = 48)	Rehospitalization − (n = 40)	*p* Value
Age (years)	56.9 ± 10	55 ± 10	59 ± 10	0.09
Men, n (%)	74 (84.6%)	42 (88.3%)	32 (80%)	0.3
BSA (m^2^)	1.95 ± 0.2	1.97 ± 0.2	1.92 ± 0.2	0.3
NYHA class (II; III; IV), n (%)	16 (19%) 29 (33%) 38 (44%)	5 (11%) 17 (37%) 24 (51%)	11 (28.5%) 11 (28.5%) 15 (37%)	0.02
NT-proBNP	8576 ± 9088	11,273 ± 10,211	4426 ± 4238	0.01
Heart rate (bpm)	78 ± 15	78 ± 14	79 ± 15	0.1
SBP (mmHg)	113 ± 11	110 ± 11	117 ± 10	0.01
DBP (mmHg)	69 ± 7	67 ± 7	71 ± 8	0.03
HTN, n (%)	25 (29%)	11(23%)	15 (37%)	0.2
DM, n (%)	30 (34%)	14 (30%)	16 (40%)	0.4
Dyslipidemia, n (%)	59 (67%)	28 (58%)	32 (80%)	0.05
Active smoking, n (%)	24 (27%)	14 (30%)	11 (29%)	0.6

Categorical variables are expressed numerically and in percentages. Continuous variables are expressed as mean and standard deviation.

**Table 2:** Pathogenic and symptomatic treatment for HF.

Drug Class	All Pts (n = 88)	Rehospitalization + (n = 48)	Rehospitalization − (n = 40)	*p* Value
Betablocker	84 (96%)	45 (95%)	39 (97%)	>0.05
ACEI/ARB	21 (24%)	8 (16%)	13 (34%)	
ARNI	43 (49%)	25 (53%)	17 (43%)	
SGLT2 inhibitor	38 (43%)	21 (44%)	16 (42%)	
MRA	79 (90%)	42 (88%)	36 (91%)	
Diuretic	83 (94%)	44 (93%)	37 (94%)	

Categorical variables are expressed numerically and in percentages. Continuous variables are expressed as mean and standard deviation.

**Table 3:** Echocardiographic data for left heart parameters.

Variable	Echo Technique	All Pts (n = 88)	Rehospitalization + (n = 48)	Rehospitalization − (n = 40)	*p* Value
E/E’	PWD, TDI PW	13.5 ± 7	15 ± 9	12 ± 6	0.2
LV GLS (%)	2DSTE	7.2 ± 3	6.4 ± 3	8.1 ± 3	0.02
LASr BP (%)	2DSTE	9.5 ± 6	8.8 ± 6	10.3 ± 6	0.2
LVEDVi (mL/m^2^)	3DE	129.2 ± 53	138.5 ± 59	120 ± 46	0.2
LVESVi (mL/m^2^)	3DE	91.4 ± 43	101 ± 47	81 ± 37	0.1
LVSVi (mL/m^2^)	3DE	32.9 ± 9	32.6 ± 7	33.2 ± 11	0.8
LVEF (%)	3DE	30.2 ± 9	26.8 ± 8	33.1 ± 10	0.01
maxLAVi (mL/m^2^)	3DE	50.3 ± 15	54.1 ± 16	46.6 ± 12	0.08
minLAVi (mL/m^2^)	3DE	39.9 ± 15	44.3 ± 16	35.3 ± 13	0.04
LAEF (%)	3DE	23.4 ± 13	18.7 ± 19	27.6 ± 14	0.01

**Table 4:** Echocardiographic data for right heart parameters.

Variable	Echo Technique	All Pts (n = 88)	Rehospitalization + (n = 48)	Rehospitalization − (n = 40)	*p* Value
TAPSE (mm)	M mode	16.5 ± 3	15.2 ± 3	17.9 ± 4	0.001
RVFAC (%)	2DE	34.9 ± 9	31 ± 10	39.7 ±6	<0.001
RVFWLS (%)	2DSTE	15.1 ± 6	13.1 ± 6	17.6 ± 5	0.001
RASr (%)	2DSTE	15.5 ± 11	12.5 ± 10	19.3 ± 10	0.009
RVEDVi (mL/m^2^)	3DE	61 ± 28	70.3 ± 28	50.7 ± 24	0.01
RVESVi (mL/m^2^)	3DE	37.9 ± 21	47.4 ± 21	27.1 ± 15	0.001
RVSVi (mL/m^2^)	3DE	23 ± 9	22.7 ± 8	23.4 ± 11	0.8
RVEF (%)	3DE	41.2 ± 9	36.7 ± 9	46.7 ± 7	<0.001
maxRAVi (mL/m^2^)	3DE	39.7 ± 19	46.9 ± 21	32.2 ± 13	0.008
minRAVi (mL/m^2^)	3DE	30.3 ± 17	37 ± 19	23.5 ± 11	0.006
RAEF (%)	3DE	26.5 ± 13	22.3 ± 12	30.3 ± 13	0.02
PASP (mmHg)	CWD, 2DE	34 ± 14	40 ± 15	28 ± 14	<0.001

**Table 5:** Surrogate parameter values of RV-PA coupling.

Variable	All Pts (n = 88)	Rehospitalization + (n = 48)	Rehospitalization − (n = 40)	*p* Value
TAPSE/PASP (mm/mmHg)	0.59 ± 0.3	0.46 ± 0.2	0.82 ± 0.4	0.002
RVFAC/PASP (%mmHg)	1.37 ± 0.8	1 ± 0.8	1.79 ± 0.9	0.001
RVFWLS/PASP (%mmHg)	0.57 ± 0.4	0.44 ± 0.4	0.81 ± 0.5	0.002
RVEF/PASP (%mmHg)	2.55 ± 2.3	1.1 ± 0.8	2.1 ± 1	<0.001

**Table 6:** Univariate and multivariate Cox regression analysis for predictors of rehospitalization for decompensated heart failure.

Parameter	Univariate Analysis		Multivariate Analysis
	HR (95% CI)	*p* Value	*p* Value
LV GLS	0.89 (0.79–0.99)	0.045	
LVEF	0.95 (0.92–0.99)	0.043	
TAPSE	0.88 (0.81–0.96)	0.007	
RVFAC	0.93 (0.89–0.96)	<0.001	
RVFWLS	0.90 (0.85–0.96)	0.001	
RVEF	0.92 (0.89–0.95)	<0.001	
TAPSE/PASP	0.14 (0.04–0.4)	0.001	
RVFAC/PASP	0.42 (0.26–0.69)	<0.001	
RVFWLS/PASP	0.21 (0.08–0.5)	0.002	0.002
RVEF/PASP	0.45 (0.29–0.69)	<0.001	0.001

**Table 7:** Clinical and laboratory characteristics of patients according to mortality status.

Variable	Died (n = 20)	Alive (n = 68)	*p* Value
Age (years)	59.9 ± 10	56.1 ± 11	>0.05
Men, n (%)	20 (100%)	55 (80.9%)	
BSA (m^2^)	1.96 ± 0.2	1.94 ± 0.2	
NT-proBNP	13,115 ± 9865	7567 ± 8783	0.01
NYHA class (II; III; IV), n (%)	73 ± 13	80 ± 15	>0.05
Heart rate (bpm)	110 ± 13	114 ± 11	
SBP (mmHg)	69 ± 8	69 ± 7	
DBP (mmHg)	30%	28%	
HTN, n (%)	26%	36%	
Diabetes, n (%)	71%	53%	
Dyslipidemia, n (%)	40%	24%	

Categorical variables are expressed numerically and in percentages. Continuous variables are expressed as mean and standard deviation.

**Table 8:** Comparison of non-invasive RV–PA coupling parameters according to all-cause mortality status.

Variable	Died (n = 20)	Alive (n = 68)	*p* Value
TAPSE/PASP (mm/mmHg)	0.61 ± 0.4	0.69 ± 0.5	0.5
RVFAC/PASP (%/mmHg)	1.31 ± 0,9	1.54 ± 1	0.4
RVFWLS/PASP (%/mmHg)	0.60 ± 0.5	0.61 ± 0.4	0.9
RVEF/PASP (%/mmHg)	1.55 ± 1	1.75 ± 1.1	0.5

## Data Availability

Data is available on request.

## Supplementary Materials

The following supporting information can be downloaded at: https://www.mdpi.com/article/10.3390/jcm15062239/s1, Table S1. Spearman correlation coefficients between RV parameters; Table S2. Variance inflation factors (VIF) for echocardiographic variables inluded in multivariate models.

## Abbreviations

The following abbreviations are used in this manuscript:

AUC	Area under the curve
BSA	Body surface area
CWD	Continuous-wave Doppler
DBP	Diastolic blood pressure
DCM	Dilated cardiomyopathy
Ea	Arterial elastance
Ees	End-systolic elastance
GLS	Global longitudinal strain
HF	Heart failure
HR	Hazard ratio
HTN	Hypertension
IVC	Inferior vena cava
LA	Left atrium
LVEF	Left ventricular ejection fraction
LVEDV	Left ventricular end-diastolic volume
LVESV	Left ventricular end-systolic volume
NT-proBNP	N-terminal pro-B-type natriuretic peptide
NYHA	New York Heart Association
PA	Pulmonary artery
PAH	Pulmonary arterial hypertension
PASP	Pulmonary artery systolic pressure
PH	Pulmonary hypertension
RA	Right atrium
ROC	Receiver operating characteristic
RV	Right ventricle
RVFAC	Right ventricular fractional area change
RVFWLS	Right ventricular free wall longitudinal strain
RVEF	Right ventricular ejection fraction
RVSV	Right ventricular stroke volume
SBP	Systolic blood pressure
TAPSE	Tricuspid annular plane systolic excursion
TTE	Transthoracic echocardiography
2DE	Two-dimensional echocardiography
2DSTE	Two-dimensional speckle-tracking echocardiography
3DE	Three-dimensional echocardiography

## References

[1] Pueschner A., Chattranukulchai P., Heitner J.F., Shah D.J., Hayes B., Rehwald W., Parker M.A., Kim H.W., Judd R.M., Kim R.J. (2017). The Prevalence, Correlates, and Impact on Cardiac Mortality of Right Ventricular Dysfunction in Nonischemic Cardiomyopathy. JACC Cardiovasc. Imaging.

[2] Schmeißer A., Rauwolf T., Groscheck T., Fischbach K., Kropf S., Luani B., Tanev I., Hansen M., Meißler S., Schäfer K. (2021). Predictors and Prognosis of Right Ventricular Function in Pulmonary Hypertension Due to Heart Failure with Reduced Ejection Fraction. ESC Heart Fail..

[3] La Vecchia L., Zanolla L., Varotto L., Bonanno C., Spadaro G.L., Ometto R., Fontanelli A. (2001). Reduced Right Ventricular Ejection Fraction as a Marker for Idiopathic Dilated Cardiomyopathy Compared with Ischemic Left Ventricular Dysfunction. Am. Heart J..

[4] Venner C., Selton-Suty C., Huttin O., Erpelding M.-L., Aliot E., Juillière Y. (2016). Right Ventricular Dysfunction in Patients with Idiopathic Dilated Cardiomyopathy: Prognostic Value and Predictive Factors. Arch. Cardiovasc. Dis..

[5] Gulati A., Ismail T.F., Jabbour A., Alpendurada F., Guha K., Ismail N.A., Raza S., Khwaja J., Brown T.D.H., Morarji K. (2013). The Prevalence and Prognostic Significance of Right Ventricular Systolic Dysfunction in Nonischemic Dilated Cardiomyopathy. Circulation.

[6] Sanz J., Sánchez-Quintana D., Bossone E., Bogaard H.J., Naeije R. (2019). Anatomy, Function, and Dysfunction of the Right Ventricle: JACC State-of-the-Art Review. J. Am. Coll. Cardiol..

[7] Iovănescu M.L., Florescu D.R., Marcu A.S., Donoiu I., Militaru S., Florescu C., Istrătoaie O., Militaru C. (2022). The Dysfunctional Right Ventricle in Dilated Cardiomyopathies: Looking from the Right Point of View. J. Cardiovasc. Dev. Dis..

[8] Dandel M., Hetzer R. (2016). Echocardiographic Assessment of the Right Ventricle: Impact of the Distinctly Load Dependency of Its Size, Geometry and Performance. Int. J. Cardiol..

[9] Monge García M.I., Santos A. (2020). Understanding Ventriculo-Arterial Coupling. Ann. Transl. Med..

[10] Ikonomidis I., Aboyans V., Blacher J., Brodmann M., Brutsaert D.L., Chirinos J.A., De Carlo M., Delgado V., Lancellotti P., Lekakis J. (2019). The Role of Ventricular-Arterial Coupling in Cardiac Disease and Heart Failure: Assessment, Clinical Implications and Therapeutic Interventions. A Consensus Document of the European Society of Cardiology Working Group on Aorta & Peripheral Vascular Diseases, European Association of Cardiovascular Imaging, and Heart Failure Association. Eur. J. Heart Fail..

[11] Tello K., Wan J., Dalmer A., Vanderpool R., Ghofrani H.A., Naeije R., Roller F., Mohajerani E., Seeger W., Herberg U. (2019). Validation of the Tricuspid Annular Plane Systolic Excursion/Systolic Pulmonary Artery Pressure Ratio for the Assessment of Right Ventricular-Arterial Coupling in Severe Pulmonary Hypertension. Circ. Cardiovasc. Imaging.

[12] Arbelo E., Protonotarios A., Gimeno J.R., Arbustini E., Barriales-Villa R., Basso C., Bezzina C.R., Biagini E., Blom N.A., de Boer R.A. (2023). 2023 ESC Guidelines for the Management of Cardiomyopathies. Eur. Heart J..

[13] Lang R.M., Badano L.P., Mor-avi V., Afilalo J., Armstrong A., Ernande L., Flachskampf F.A., Foster E., Goldstein S.A., Kuznetsova T. (2015). Recommendations for Cardiac Chamber Quantification by Echocardiography in Adults: An Update from the American Society of Echocardiography and the European Association of Cardiovascular Imaging. Eur. Heart J. Cardiovasc. Imaging.

[14] Pinto Y.M., Elliott P.M., Arbustini E., Adler Y., Anastasakis A., Böhm M., Duboc D., Gimeno J., de Groote P., Imazio M. (2016). Proposal for a Revised Definition of Dilated Cardiomyopathy, Hypokinetic Non-Dilated Cardiomyopathy, and Its Implications for Clinical Practice: A Position Statement of the ESC Working Group on Myocardial and Pericardial Diseases. Eur. Heart J..

[15] Ilardi F., D’Andrea A., D’Ascenzi F., Bandera F., Benfari G., Esposito R., Malagoli A., Mandoli G.E., Santoro C., Russo V. (2021). Myocardial Work by Echocardiography: Principles and Applications in Clinical Practice. J. Clin. Med..

[16] Badano L.P., Muraru D., Parati G., Haugaa K., Voigt J.-U. (2020). How to Do Right Ventricular Strain. Eur. Heart J. Cardiovasc. Imaging.

[17] Badano L.P., Addetia K., Pontone G., Torlasco C., Lang R.M., Parati G., Muraru D. (2020). Advanced Imaging of Right Ventricular Anatomy and Function. Heart.

[18] Rosenkranz S., Gibbs J.S.R., Wachter R., De Marco T., Vonk-Noordegraaf A., Vachiéry J.L. (2016). Left Ventricular Heart Failure and Pulmonary Hypertension. Eur. Heart J..

[19] Al-Omary M.S., Sugito S., Boyle A.J., Sverdlov A.L., Collins N.J. (2020). Pulmonary Hypertension Due to Left Heart Disease. Hypertension.

[20] Vachiéry J.L., Adir Y., Barberà J.A., Champion H., Coghlan J.G., Cottin V., Marco T., Galiè N., Ghio S., Gibbs J.S.R. (2013). Pulmonary Hypertension Due to Left Heart Diseases. J. Am. Coll. Cardiol..

[21] Antigny F., Mercier O., Humbert M., Sabourin J. (2020). Excitation-Contraction Coupling and Relaxation Alteration in Right Ventricular Remodelling Caused by Pulmonary Arterial Hypertension. Arch. Cardiovasc. Dis..

[22] Vonk Noordegraaf A., Westerhof B.E., Westerhof N. (2017). The Relationship Between the Right Ventricle and Its Load in Pulmonary Hypertension. J. Am. Coll. Cardiol..

[23] Nagata Y., Wu V.C.-C., Kado Y., Otani K., Lin F.-C., Otsuji Y., Negishi K., Takeuchi M. (2017). Prognostic Value of Right Ventricular Ejection Fraction Assessed by Transthoracic 3D Echocardiography. Circ. Cardiovasc. Imaging.

[24] Merlo M., Gobbo M., Stolfo D., Losurdo P., Ramani F., Barbati G., Pivetta A., Di Lenarda A., Anzini M., Gigli M. (2016). The Prognostic Impact of the Evolution of RV Function in Idiopathic DCM. JACC Cardiovasc. Imaging.

[25] Rudski L.G., Lai W.W., Afilalo J., Hua L., Handschumacher M.D., Chandrasekaran K., Solomon S.D., Louie E.K., Schiller N.B. (2010). Guidelines for the Echocardiographic Assessment of the Right Heart in Adults: A Report from the American Society of Echocardiography. J. Am. Soc. Echocardiogr..

[26] Zhao R., Li D., Zuo P., Bai R., Zhou Q., Fan J., Li C., Wang L., Yang X. (2014). Influences of Age, Gender, and Circadian Rhythm on Deceleration Capacity in Subjects without Evident Heart Diseases. Ann. Noninvasive Electrocardiol..

[27] Smolarek D., Gruchała M., Sobiczewski W. (2017). Echocardiographic Evaluation of Right Ventricular Systolic Function: The Traditional and Innovative Approach. Cardiol. J..

[28] Vîjîiac A., Onciul S., Guzu C., Verinceanu V., Bătăilă V., Deaconu S., Scărlătescu A., Zamfir D., Petre I., Onuţ R. (2021). The Prognostic Value of Right Ventricular Longitudinal Strain and 3D Ejection Fraction in Patients with Dilated Cardiomyopathy. Int. J. Cardiovasc. Imaging.

[29] Bosch L., Lam C.S.P., Gong L., Chan S.P., Sim D., Yeo D., Jaufeerally F., Leong K.T.G., Ong H.Y., Ng T.P. (2017). Right Ventricular Dysfunction in Left-Sided Heart Failure with Preserved versus Reduced Ejection Fraction. Eur. J. Heart Fail..

[30] Bragança B., Trêpa M., Santos R., Silveira I., Fontes-Oliveira M., Sousa M.J., Reis H., Torres S., Santos M. (2020). Echocardiographic Assessment of Right Ventriculo-Arterial Coupling: Clinical Correlates and Prognostic Impact in Heart Failure Patients Undergoing Cardiac Resynchronization Therapy. J. Cardiovasc. Imaging.

[31] Guazzi M., Naeije R., Arena R., Corrà U., Ghio S., Forfia P., Rossi A., Cahalin L.P., Bandera F., Temporelli P. (2015). Echocardiography of Right Ventriculoarterial Coupling Combined with Cardiopulmonary Exercise Testing to Predict Outcome in Heart Failure. Chest.

[32] Schmeisser A., Rauwolf T., Groscheck T., Kropf S., Luani B., Tanev I., Hansen M., Meißler S., Steendijk P., Braun-Dullaeus R.C. (2021). Pressure-Volume Loop Validation of TAPSE/PASP for Right Ventricular Arterial Coupling in Heart Failure with Pulmonary Hypertension. Eur. Heart J. Cardiovasc. Imaging.

[33] Giusca S., Dambrauskaite V., Scheurwegs C., D’hooge J., Claus P., Herbots L., Magro M., Rademakers F., Meyns B., Delcroix M. (2010). Deformation Imaging Describes Right Ventricular Function Better than Longitudinal Displacement of the Tricuspid Ring. Heart.

[34] Gavazzoni M., Badano L.P., Vizzardi E., Raddino R., Genovese D., Taramasso M., Sciatti E., Palermo C., Metra M., Muraru D. (2020). Prognostic Value of Right Ventricular Free Wall Longitudinal Strain in a Large Cohort of Outpatients with Left-Side Heart Disease. Eur. Heart J. Cardiovasc. Imaging.

[35] Houard L., Benaets M.-B., de Meester de Ravenstein C., Rousseau M.F., Ahn S.A., Amzulescu M.-S., Roy C., Slimani A., Vancraeynest D., Pasquet A. (2019). Additional Prognostic Value of 2D Right Ventricular Speckle-Tracking Strain for Prediction of Survival in Heart Failure and Reduced Ejection Fraction: A Comparative Study With Cardiac Magnetic Resonance. JACC Cardiovasc. Imaging.

[36] Carluccio E., Biagioli P., Alunni G., Murrone A., Zuchi C., Coiro S., Riccini C., Mengoni A., D’Antonio A., Ambrosio G. (2018). Prognostic Value of Right Ventricular Dysfunction in Heart Failure with Reduced Ejection Fraction: Superiority of Longitudinal Strain Over Tricuspid Annular Plane Systolic Excursion. Circ. Cardiovasc. Imaging.

[37] Ünlü S., Bézy S., Cvijic M., Duchenne J., Delcroix M., Voigt J.-U. (2023). Right Ventricular Strain Related to Pulmonary Artery Pressure Predicts Clinical Outcome in Patients with Pulmonary Arterial Hypertension. Eur. Heart J. Cardiovasc. Imaging.

[38] Iacoviello M., Monitillo F., Citarelli G., Leone M., Grande D., Antoncecchi V., Rizzo C., Terlizzese P., Romito R., Caldarola P. (2017). Right Ventriculo-Arterial Coupling Assessed by Two-Dimensional Strain: A New Parameter of Right Ventricular Function Independently Associated with Prognosis in Chronic Heart Failure Patients. Int. J. Cardiol..

[39] Hădăreanu C.-D., Hădăreanu D.-R., Toader D.-M., Iovănescu M.-L., Florescu C., Raicea V.-C., Donoiu I. (2025). Prognostic Value of the Ratio between Right Ventricular Free Wall Longitudinal Strain and Systolic Pulmonary Artery Pressure in Patients with Heart Failure with Reduced Ejection Fraction and Ventricular Secondary Mitral Regurgitation. Front. Cardiovasc. Med..

[40] Sugeng L., Mor-Avi V., Weinert L., Niel J., Ebner C., Steringer-Mascherbauer R., Bartolles R., Baumann R., Schummers G., Lang R.M. (2010). Multimodality Comparison of Quantitative Volumetric Analysis of the Right Ventricle. JACC Cardiovasc. Imaging.

[41] D’Andrea A., Gravino R., Riegler L., Salerno G., Scarafile R., Romano M., Cuomo S., Del Viscovo L., Ferrara I., De Rimini M.L. (2011). Right Ventricular Ejection Fraction and Left Ventricular Dyssynchrony by 3D Echo Correlate with Functional Impairment in Patients with Dilated Cardiomyopathy. J. Card. Fail..

[42] Sabe M.A., Sabe S.A., Kusunose K., Flamm S.D., Griffin B.P., Kwon D.H. (2016). Predictors and Prognostic Significance of Right Ventricular Ejection Fraction in Patients with Ischemic Cardiomyopathy. Circulation.

[43] Nochioka K., Querejeta Roca G., Claggett B., Biering-Sørensen T., Matsushita K., Hung C.-L., Solomon S.D., Kitzman D., Shah A.M. (2018). Right Ventricular Function, Right Ventricular-Pulmonary Artery Coupling, and Heart Failure Risk in 4 US Communities: The Atherosclerosis Risk in Communities (ARIC) Study. JAMA Cardiol..

[44] Hsu S., Simpson C.E., Houston B.A., Wand A., Sato T., Kolb T.M., Mathai S.C., Kass D.A., Hassoun P.M., Damico R.L. (2020). Multi-Beat Right Ventricular-Arterial Coupling Predicts Clinical Worsening in Pulmonary Arterial Hypertension. J. Am. Heart Assoc..

