# Supplementary material for: Prognostic Value of Non-Invasively Determined Right Ventricular–Arterial Coupling Surrogate Parameters in Patients with Dilated Cardiomyopathy
Source: J Clin Med. 2026 Mar 16;15(6):2239. doi: 10.3390/jcm15062239 (PMC13027013; doi:10.3390/jcm15062239)
Supplement: Supplementary file 1 [file jcm-15-02239-s001.zip › jcm-4012651-supplementary.pdf]

Supplementary Table S1. Spearman correlation coefficients between RV parameters.

Variable	TAPSE	sPAP	TAPSE/PAPS	RVFAC	RVFWLS	RVEF	RVFWLS/PAPS	RVEF/PAPS
**TAPSE**	1.00	−0.19	0.65	0.31	0.36	0.37	0.35	0.09
**sPAP**	−0.19	1.00	−0.83	−0.43	−0.45	−0.43	−0.87	−0.41
**TAPSE/PAPS**	0.65	−0.83	1.00	0.38	0.45	0.43	0.76	0.40
**RVFAC**	0.31	−0.43	0.38	1.00	0.72	0.74	0.63	−0.40
**RVFWLS**	0.36	−0.45	0.45	0.72	1.00	0.71	0.81	−0.43
**RVEF**	0.37	−0.43	0.43	0.74	0.71	1.00	0.68	−0.37
**RVFWLS/PAPS**	0.35	−0.87	0.76	0.63	0.81	0.68	1.00	−0.01
**RVEF/PAPS**	0.09	−0.41	0.40	−0.40	−0.43	−0.37	−0.01	1.00

Supplementary Table S2. Variance inflation factors (VIF) for echocardiographic variables inluded in multivariate models.

Parameter	VIF
**TAPSE**	1.19
**sPAP**	1.27
**RVFAC**	2.56
**RVFWLS**	2.22
**RVEF**	3.10
